# Hepatocellular carcinoma development in diabetic patients: a nationwide survey in Japan

**DOI:** 10.1007/s00535-020-01754-z

**Published:** 2021-01-11

**Authors:** Ryosuke Tateishi, Takeshi Matsumura, Takeshi Okanoue, Toshihide Shima, Koji Uchino, Naoto Fujiwara, Takafumi Senokuchi, Kazuyoshi Kon, Takayoshi Sasako, Makiko Taniai, Takumi Kawaguchi, Hiroshi Inoue, Hirotaka Watada, Naoto Kubota, Hitoshi Shimano, Shuichi Kaneko, Etsuko Hashimoto, Sumio Watanabe, Goshi Shiota, Kohjiro Ueki, Kosuke Kashiwabara, Yutaka Matsuyama, Hideo Tanaka, Masato Kasuga, Eiichi Araki, Kazuhiko Koike, for the LUCID study investigators, Yoshiyasu Karino, Shuhei Hige, Masatomo Sekiguchi, Koji Ogawa, Hideaki Miyoshi, KyuYong Cho, Masaru Baba, Atsushi Inoue, Kazuobu Aso, Mitsuyoshi Okada, Yasuhiro Takikawa, Kei Endo, Yasushi Ishigaki, Hirobumi Togashi, Michiaki Unno, Takanori Morikawa, Hideki Katagiri, Shojiro Sawada, Hiromasa Ohira, Atsushi Takahashi, Michio Shimabukuro, Akihiro Kudo, Naomi Tanaka, Junko Mitsuhashi, Toshifumi Tokai, Yasushi Matsuzaki, Tadashi Ikegami, Masato Imai, Kou Nishikawa, Sadao Takahashi, Sumiko Nagoshi, Kosuke Maezawa, Masafumi Matsuda, Tetsuhiro Chiba, Masayuki Yokoyama, Koutaro Yokote, Yoko Hidetaka, Mitsuhiko Moriyama, Hitomi Nakamura, Midori Fujishiro, Tadakazu Hisamatsu, Kaori Nishikawa, Toshihiko Nouchi, Yuki Sakurai, Harumi Daikoku, Michinori Murayama, Yoshinori Saigusa, Takashi Matsui, Kazuhiko Koike, Ryosuke Tateishi, Naoto Kubota, Takayoshi Sasako, Hidenari Nagai, Takanori Mukozu, Takahisa Hirose, Masahiko Miyagi, Sumio Watanabe, Kazuyoshi Kon, Hirotaka Watada, Hiroaki Sato, Akira Mizuki, Masanori Miura, Yuzuru Sato, Rena Kaneko, Kumiko Hamano, Hiroko Kato, Kentaro Kikuchi, Yusuke Kajiyama, Makoto Chuma, Hiroko Ito, Hiroshi Yasuda, Nobuyuki Matsumoto, Yasushi Tanaka, Yoshio Nagai, Kento Imajo, Yasuo Terauchi, Yuzuru Ito, Shuji Terai, Hirohito Sone, Satoshi Matsunaga, Kiyoshi Furuta, Eiji Tanaka, Takeji Umemura, Mitsuhisa Komatsu, Yoshihiko Sato, Yoshinobu Hinoue, Akiko Shimizu, Terumi Takahara, Kengo Kawai, Kazuyuki Tobe, Isao Usui, Hajime Ohta, Takuya Komura, Seiichiro Kurita, Nobuyuki Toshikuni, Daisuke Koya, Ai Watanabe, Yasunari Nakamoto, Tomoyuki Nemoto, Tadashi Konoshita, Yoshimasa Kobayashi, Shin Shimoyama, Daisaku Nishimura, Masami Imoto, Sayori Nakashima, Tatsuo Mizuno, Kentaro Yoshioka, Naoto Kawabe, Suzuki Atsushi, Ayako Kakita, Yuji Kojima, Kazuya Murata, Yoshiyuki Takei, Motoo Iwasa, Yutaka Yano, Masahiko Kondo, Motozumi Okamoto, Hiroyuki Kimura, Hiroyuki Marusawa, Yuji Eso, Yoshihito Fujita, Hideki Fujii, Masaaki Inaba, Masanori Emoto, Takeshi Kurose, Takeshi Okanoue, Toshihide Shima, Shinichi Mogami, Rinako Tsuda, Emiko Nomura, Masayoshi Ohnishi, Masatoshi Kudo, Naoshi Nishida, Hiroshi Ikegami, Hiroyuki Ito, Hirokazu Oyamada, Yasutaka Nagao, Hiroshi Okada, Masahide Oshita, Tetsuyuki Yasuda, Toshifumi Ito, Nobuyuki Tatsumi, Teruhisa Morikawa, Noriko Takahara, Takumi Fukumoto, Motofumi Tanaka, Wataru Ogawa, Yushi Hirota, Seitetsu Yung, Keiji Iida, Hitoshi Yoshiji, Naotaka Shimozato, Gen Inoue, Tomonao Hirobata, Junichi Okano, Tsuyoshi Okura, Yasushi Uchida, Toshiaki Sato, Miwa Kawanaka, Takatoshi Anno, Keisuke Hino, Yasuyuki Tomiyama, Hideaki Kaneto, Shinichi Fujioka, Eri Sasaki, Tatsuaki Nakato, Keiji Tsuji, Yohji Honda, Nozomu Kamei, Mitsue Miyahara, Tsutomu Masaki, Kyoko Oura, Yoshiyasu Kisaka, Shozo Miyauchi, Isao Makino, Yoichi Hiasa, Yohei Koizumi, Osawa Haruhiko, Toshimi Hadate, Takeaki Sato, Isao Ichino, Hideyuki Nomura, Nobuyuki Yamashita, Hiroshi Maeno, Sunao Matubayashi, Kenta Motomura, Yukie Ishibashi, Makoto Ide, Yoko Moribe, Masaru Harada, Michihiko Shibata, Yosuke Okada, Keiichi Torimoto, Takuji Torimura, Takumi Kawaguchi, Yuji Tajiri, Nobuhiko Higashi, Sakae Nohara, Yasunori Kawaguchi, Toru Yoshimura, Keizo Anzai, Shinji Iwane, Kazuhiko Nakao, Naota Taura, Norio Abiru, Yoshitaka Mori, Masataka Seike, Mizuki Endo, Tadanobu Kuribayashi

**Affiliations:** 1 Department of Gastroenterology, Graduate School of Medicine, The University of Tokyo, 7-3-1 Hongo, Bunkyo-ku, Tokyo, 113-8655 Japan; 2 Department of Metabolic Medicine, Kumamoto University Faculty of Life Sciences, Kumamoto, Japan; 3 Department of Gastroenterology and Hepatology, Saiseikai Suita Hospital, Suita, Japan; 4 Department of Gastroenterology, Juntendo University School of Medicine, Tokyo, Japan; 5 Department of Diabetes and Metabolic Diseases, The University of Tokyo Graduate School of Medicine, Tokyo, Japan; 6 Institute of Gastroenterology, Department of Internal Medicine, Tokyo Women’s Medical University, Tokyo, Japan; 7 Division of Gastroenterology, Department of Medicine, Kurume University School of Medicine, Kurume, Japan; 8 Metabolism and Nutrition Research Unit, Kanazawa University Institute for Frontier Science Initiative, Kanazawa, Japan; 9 Department of Medicine, Metabolism and Endocrinology, Juntendo University School of Medicine, Tokyo, Japan; 10 Department of Internal Medicine, Metabolism and Endocrinology, Tsukuba University, Tsukuba, Japan; 11 Department of Gastroenterology, Kanazawa University Graduate School of Medical Science, Kanazawa, Japan; 12 Division of Molecular and Genetic Medicine, Institute of Regenerative Medicine and Biofunction, Graduate School of Medicine, Tottori University, Yonago, Japan; 13 Diabetes Research Center, Research Institute, National Center for Global Health and Medicine, Tokyo, Japan; 14 Department of Biostatistics, The University of Tokyo Graduate School of Medicine, Tokyo, Japan; 15 Fujiidera Public Health Center, Fujiidera, Japan; 16 The Institute for Adult Diseases, Asahi Life Foundation, Tokyo, Japan; 17 Department of Molecular Sciences on Diabetes, Graduate School of Medicine, The University of Tokyo, Tokyo, Japan

**Keywords:** Hepatocellular carcinoma, Type 2 diabetes mellitus, FIB-4 index

## Abstract

**Background:**

Although type 2 diabetes mellitus (T2DM) is a known risk factor for hepatocellular carcinoma (HCC) development, the annual incidence in diabetes patients is far below the threshold of efficient surveillance. This study aimed to elucidate the risk factors for HCC in diabetic patients and to determine the best criteria to identify surveillance candidates.

**Methods:**

The study included 239 patients with T2DM who were diagnosed with non-viral HCC between 2010 and 2015, with ≥ 5 years of follow-up at diabetes clinics of 81 teaching hospitals in Japan before HCC diagnosis, and 3277 non-HCC T2DM patients from a prospective cohort study, as controls. Clinical data at the time of and 5 years before HCC diagnosis were collected.

**Results:**

The mean patient age at HCC diagnosis was approximately 73 years, and 80% of the patients were male. The proportion of patients with insulin use increased, whereas the body mass index (BMI), proportion of patients with fatty liver, fasting glucose levels, and hemoglobin A1c (HbA1c) levels decreased significantly in 5 years. In the cohort study, 18 patients developed HCC during the mean follow-up period of 4.7 years with an annual incidence of 0.11%. Multivariate logistic regression analyses showed that the FIB-4 index was an outstanding predictor of HCC development along with male sex, presence of hypertension, lower HbA1c and albumin levels, and higher BMI and gamma-glutamyl transpeptidase levels. Receiver-operating characteristic analyses showed that a FIB-4 cut-off value of 3.61 could help identify high-risk patients, with a corresponding annual HCC incidence rate of 1.1%.

**Conclusion:**

A simple calculation of the FIB-4 index in diabetes clinics can be the first step toward surveillance of HCC with a non-viral etiology.

## Introduction

Globally, liver cancer is the seventh most common cancer and the fourth most common cause of death [1]. In hepatocellular carcinoma (HCC), which accounts for 70% to 85% of all liver cancers [2], chronic viral hepatitis plays a major role; approximately 77% of liver cancer cases were estimated to be attributable to either hepatitis B virus (HBV) or hepatitis C virus (HCV) infection in 2008 [3].

While HCC is a typical example of virus-related cancer, it is also strongly related to lifestyle. Chronic alcoholism is a classical risk factor [4]. Growing evidence suggests that obesity and diabetes increase various cancer risks [5–9], and the liver is one of the organs upon which obesity and obesity-associated conditions have the largest impact [5, 8, 10, 11]. Obesity and diabetes represent the largest risk factors for liver cancer development in the United States [12], and the increase in the incidence of HCC was the highest among all types of cancers between 2003 and 2012 [13].

Due to changes in dietary habits in the last four decades, the proportion of overweight males has continued to increase for all ages and in elderly females in Japan. The increase in the overweight and obese population has resulted in an increased number of patients with diabetes mellitus and fatty liver [14, 15]. The Japan Diabetes Society (JDS) conducted a nationwide survey investigating causes of death in 45,708 diseased Japanese patients with diabetes from 2001–2010 [16]. According to the report, the most frequent cause of death was malignant neoplasia (38.3%), followed by infections (17.0%), and then vascular diseases (14.9%). The liver was the second commonest site of cancer-related death after the lung in Japanese diabetic patients, whereas it ranked fifth in the general Japanese population [17]. We also reported that the incidence of HCC with a non-viral etiology increased rapidly between 1991 and 2015 in Japan from a nationwide survey including more than 7000 patients with non-viral etiology [18]. In that report, we concluded that the increase in the rate of obesity among Japanese males over the last three decades has contributed to the increase based on the larger proportion of obese and diabetic patients in the cohort.

International guidelines for HCC recommend surveillance for HCC in cirrhotic patients [19–21]. However, the precise definition of at-risk population is still controversial. Generally, the definition should be based on the incidence of HCC and potential therapeutic options to be applied. Some reports suggested that an annual incidence of 1.5–2.0% would warrant the cost-effectiveness of HCC surveillance. According to a large-scale case–control study using the Surveillance Epidemiology and End-Results Program (SEER) database, diabetes carries a threefold increase in the risk of HCC, which is much lower than the 24-fold increased risk of HBV and HCV [20, 22]. The estimated annual incidence of HCC development in diabetic patients is less than 0.1% [23], far below the threshold of efficient surveillance. To elucidate the risk factors for HCC in Japanese diabetic patients and provide the best criteria for surveillance candidates, we conducted a nation-wide survey.

## Patients and methods

### Study protocol

This study was conducted as a collaborative project of Japan Society of Hepatology (JSH) and JDS to elucidate the actual profile of hepatocellular carcinoma in diabetes clinics (LUCID). An invitation letter for study participation was sent to 333 hospitals that were certified as education hospitals by both JSH and JDS. The study protocol was approved by the University of Tokyo Medical Research Center Ethics Committee (approval number 11336) and the Institutional Review Board or Ethics Committee of each participating institution. The requirement for informed consent was waived because of the retrospective design. This study was registered with the University Hospital Medical Information Network (UMIN) Clinical Trial Registry (UMIN000026246).

### Patients with HCC in diabetes clinics

Patients with type 2 diabetes mellitus (T2DM) were eligible if they were initially diagnosed with HCC at participating hospitals between 2010 and 2015, were negative for both hepatitis B surface antigen (HBsAg) and anti-HCV antibody, and had been followed up at a diabetes clinic at the same hospital for at least 5 years before the diagnosis of HCC. HCC was diagnosed pathologically or using imaging criteria on the basis of the Japanese Clinical Practice Guidelines. Hyperattenuation during the arterial phase with washout during the late phase on dynamic CT or dynamic MRI images was regarded as a specific finding [24].

### Data collection

**Fig. 1:**
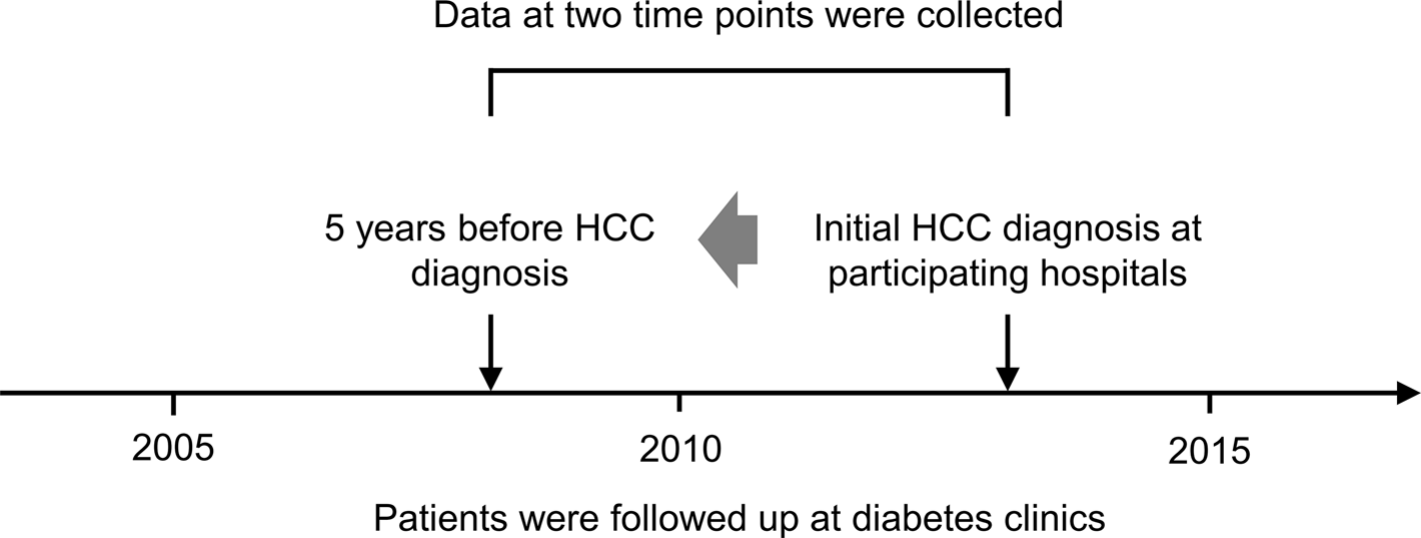
Data collection. Patients are enrolled if they were followed-up at the same diabetes clinic for at least 5 years. Data at the time of diagnosis and 5 years before the initial diagnosis of HCC are collected. HCC, hepatocellular carcinoma

The patients were registered via an electronic data capture system designed by the investigators. We collected data at two points: at the initial diagnosis of HCC and 5 years before the diagnosis of HCC (Fig. 1). The following patient characteristics were collected: age, sex, body height, body weight, daily alcohol consumption, calendar year of the initial diagnosis of diabetes, type of diabetes, family history of diabetes, medical comorbidities including hypertension, dyslipidemia, and fatty liver, diabetic complications, laboratory data, tumor characteristics, and treatment modalities. The body mass index (BMI), Child–Turcotte–Pugh (CTP) score [25], aspartate aminotransferase (AST) to platelet ratio index (APRI) [26], and fibrosis-4 (FIB-4) index [27] were calculated automatically using the obtained data. The formulae for APRI and FIB-4 were as follows.

$$ {\text{APRI}} = \frac{{{\text{AST}}\left( {/{\text{ULN}}} \right)}}{{{\text{Platelet count}} \left( {10^{9} /L} \right)}} \times 100 $$

$$ {\text{FIB}} - 4 = \frac{{{\text{Age}}\left( y \right) \times {\text{AST}}\left( {U/L} \right)}}{{{\text{Platelet count}} \left( {10^{9} /L} \right) \times \sqrt {{\text{ALT}}\left( {U/L} \right)} }} $$

### Cohort study

We utilized a pre-existing Japanese nationwide cohort of diabetic patients that enrolled patients with T2DM who visited diabetes clinics at nine participating hospitals from 2008 to 2009. A follow-up survey was conducted in 2015 to collect data at 5 years, including information regarding HCC development. The detailed protocol of the study was described elsewhere [28, 29].

We retrieved data from the database by excluding the following patients: those who did not have follow-up data including vital status, missing values in age, or with chronic hepatitis B or C. From April 1, 2012, JDS officially started to use the National Glycohemoglobin Standardization Program (NGSP) value of glycated hemoglobin (HbA1c) instead of the original JDS value. As the HbA1c value in the cohort study was obtained using the JDS value, the value was converted according to the following formula.

$$ {\text{HbA1c}}\% \left( {{\text{NGSP}}} \right) = 1.02 \times {\text{HBA1c}}\% \left( {{\text{JDS}}} \right) + 0.25\% $$

### Statistical analysis

Data were expressed as mean ± standard deviation when they were approximately normally distributed or otherwise as median with 25th–75th percentiles for continuous variables and numbers with percentages for categorical variables.

The Student *t* test or the Wilcoxon rank-sum test was used for comparisons between two continuous variables. For paired continuous variables, the paired Student’s *t *test or the Wilcoxon signed-rank test was used. Differences in the distribution at two time points were assessed using McNemar’s test. A logistic regression was used to assess the risk factors for HCC development. We used a receiver operating characteristic (ROC) curve for the assessment of sensitivity, specificity, and likelihood ratio for the positive results of the FIB-4 index. Estimated 5-year HCC rates according to FIB-4 index values were calculated on the basis of Bayes’ theorem using annual HCC incidence from the cohort study.

All statistical analyses were performed using SAS software (version 9.4, SAS Institute, Cary, NC) or R software (ver. 3.2.3; R Development Core Team, Vienna, Austria). All tests were two-sided, and *P* values < 0.05 were considered to indicate statistical significance.

## Results

### HCC patient profile

From 272 diabetic patients who were diagnosed at 81 participating hospitals during the study period, we enrolled 239 by excluding those with type 1 diabetes (*N* = 3), missing values for the exact date of the initial and last visits and HCC diagnosis (*N* = 7), unknown duration of diabetes (*N* = 6), hepatitis C status (*N* = 4), fasting glucose (*N* = 3), tumor size or number (*N* = 7), and presence of vascular invasion and extrahepatic metastasis (N = 3) (Appendix Fig. 5).

Baseline characteristics of the HCC patients with T2DM 5 years before and at the time of HCC diagnosis are shown in Table 1. The mean patient age at HCC diagnosis was approximately 73 years, and 80% of the patients were male. The median interval between the initial diagnosis of diabetes and HCC was 12 years. During the follow-up period of 5 years, the proportion of patients with insulin use increased while BMI and the proportion of patients with fatty liver, fasting glucose, and HbA1c decreased significantly.

**Table 1:** Patient characteristics 5 years before and at the time of HCC diagnosis (*N* = 239)

	Five years before HCC diagnosis	At the time of HCC diagnosis	*P*
Follow-up period, years	5.00 ± 0.33	–	
Age, years	67.8 ± 8.8	72.8 ± 8.8	
Male, *n* (%)	191 (79.9)	–	
Diabetes duration, years	7 (3–14)	12 (8–19)	
Diabetic complications, *n* (%)	117 (49.0)	–	
Alcohol consumption, g/day			
≤ 20, *n* (%)	159 (66.5)	–	
> 20 and < 60, *n* (%)	41 (17.2)	–	
≥ 60, *n* (%)	39 (16.3)	–	
BMI, kg/m^2^	26.0 ± 4.5	25.5 ± 4.8	0.01
Diabetes treatment			0.001
Diet only, *n* (%)	23 (9.6)	18 (7.5)	
Oral antidiabetic, *n* (%)	145 (60.7)	133 (55.6)	
Insulin, *n* (%)	71 (29.7)	88 (36.8)	
Diabetes complications, *n* (%)	117 (49.0)	–	
Hypertension, *n* (%)	168 (70.3)	174 (72.3)	0.32
Dyslipidemia, *n* (%)	109 (45.6)	111 (46.4)	0.87
Fatty liver, *n* (%)^a^	108 (60.3)	110 (51.2)	0.03
Fasting glucose, mg/dL	143 (121–181)	132 (110–173)	0.02
HbA1c, %	7.1 (6.5–8.1)	6.9 (6.3–7.6)	< 0.0001
Total cholesterol, mg/dL	181.1 ± 34.8	–	
Albumin, g/dL	4.07 ± 0.41	3.83 ± 0.57	< 0.0001
AST, U/L^b^	36 (25–46)	37 (26–52)	0.10
ALT, U/L^c^	34 (22–47)	26 (19–41)	0.001
GGT, U/L^d^	60 (34–142)	83 (45–160)	0.08
Creatinine, mg/dL^e^	0.80 (0.68–0.98)	0.87 (0.71–1.13)	< 0.0001
Hemoglobin, mg/dL^f^	13.9 ± 1.8	12.7 ± 2.2	< 0.0001
Platelet count, 10^4^/µL	15.5 (11.7–19.6)	14.6 (10.1–20.0)	0.17
APRI^g^	0.62 (0.38–1.10)	0.73 (0.41–1.28)	0.008
FIB-4^ h^	2.73 (1.90–4.30)	3.74 (2.43–5.76)	< 0.0001
HCC characteristics			
Maximal tumor size, cm	–	3.5 (2.0–6.5)	
Maximal tumor ≤ 3 cm, *n* (%)	–	110 (46.0)	
Single tumor, *n* (%)	–	141 (59.0)	
Vascular invasion, *n* (%)	–	36 (15.1)	
Extrahepatic metastasis, *n* (%)	–	20 (8.4)	
Within Milan criteria		123 (51.0)	
AFP ≥ 20 ng/mL, *n* (%)	–	84 (36.1)	
DCP ≥ 100 mAU/mL, *n* (%)^h^	–	120 (53.3)	
HCC treatment			
Resection, *n* (%)	-	62 (25.9)	
Ablation, *n* (%)	–	68 (28.5)	
TACE, *n* (%)	–	117 (49.0)	
Systemic therapy, *n* (%)	–	8 (3.3)	
Radiation therapy, *n* (%)	–	3 (1.3)	
Supportive care, *n* (%)		24 (10.0)	

Data are expressed as mean ± standard deviation when approximately normally distributed or otherwise median (25th–75th percentiles) for continuous variables and number (percentage) for categorical variables

Data are missing for ^a^60, ^b^7, ^c^7, ^d^19, ^e^8, ^f^9, ^g^12, and ^h^13 patients 5 years before HCC diagnosis and ^a^24, ^d^5, and ^h^4 patients at HCC diagnosis

*AST* aspartate aminotransferase; *ALT* alanine aminotransferase; *AFP* alpha-fetoprotein; *BMI* body mass index; *DCP* des-gamma-carboxy prothrombin; *FIB-4* fibrosis-4; *GGT* gamma-glutamyl transpeptidase; *APRI* AST to platelet ratio index; *HbA1c* hemoglobin A1c; *TACE* transarterial chemoembolization

Regarding the tumor characteristics, the median size of the main tumor was 3.5 cm, and approximately 60% of patients had a single tumor. A total of 123 patients (51.0%) were diagnosed within the Milan criteria (single tumor not larger than 5 cm or three or fewer tumors, none of which exceeded 3 cm without vascular invasion or extrahepatic metastasis) [30]. In 224 patients in whom the Child–Pugh class could be calculated, 176 (78.6%), 39 (17.4%), and nine (4.0%) were categorized as class A, B, and C, respectively. As a consequence, 130 patients (54.4%) underwent surgical resection or ablation.

### Cohort study

From 5,642 diabetic patients registered in the database, we enrolled 3295 after excluding type 1 diabetes mellitus (*N* = 36), those without follow-up data (*N* = 1598), with chronic hepatitis B or C (*N* = 217), with previous HCC history at enrollment (*N* = 39), and with missing values for age, exact date of initial and the last visits, and HCC status at the last visit (*N* = 457) (Appendix Fig. 6).

**Fig. 2:**
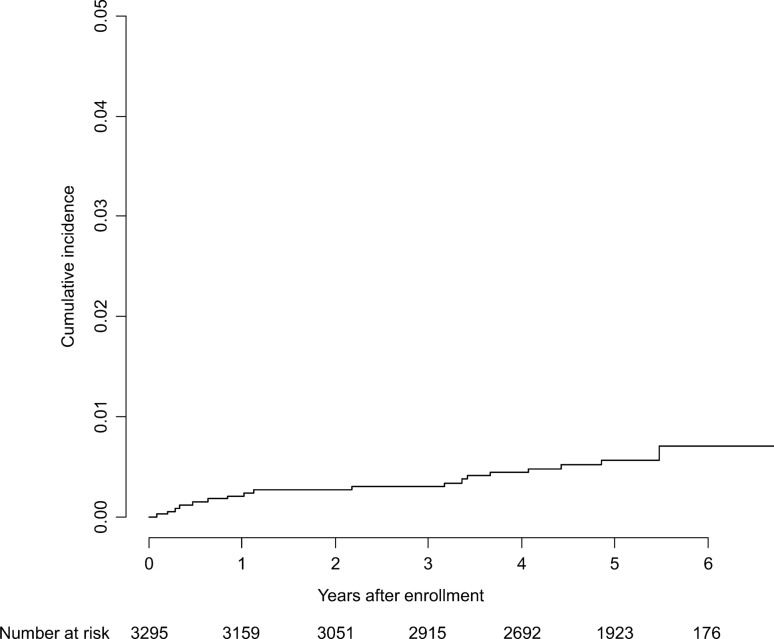
Kaplan–Meier estimate of cumulative incidence of hepatocellular carcinoma in the cohort study

During the mean follow-up period of 4.7 years, 18 patients developed HCC (Fig. 2). The annual HCC incidence calculated by the person-year method was 0.11% [95% confidence interval (CI), 0.068–0.181%]. The baseline characteristics of patients who developed HCC were similar to that of HCC patients in the current study (Table 2).

**Table 2:** Characteristics of patients in the cohort study

	HCC *N* = 18	non-HCC *N* = 3277	*P*
Age at entry, years	69.72 ± 10.3	63.3 ± 12.2	0.02
Male, *n* (%)	13 (72.2)	1887 (57.6)	0.21
Alcohol consumption, g/day^a^			< 0.0001
≤ 20, *n* (%)	9 (50.0)	2307 (80.5)	
> 20 and < 60, *n* (%)	4 (22.2)	433 (15.1)	
≥ 60, *n* (%)	5 (27.8)	126 (4.4)	
BMI, kg/m^2^	25.9 (22.5–27.0)	24.0 (21.6–26.9)	0.25
Diabetes treatment^b^			0.80
Diet only, *n* (%)	4 (22.2)	520 (16.5)	
Oral antidiabetic, *n* (%)	9 (50.0)	1626 (51.7)	
Insulin, *n* (%)	5 (27.8)	999 (31.8)	
Hypertension, *n* (%)	11 (61.1)	1680 (51.3)	0.40
Dyslipidemia, *n* (%)	6 (33.3)	1594 (48.6)	0.29
Fasting glucose, mg/dL	121 (112–138)	144 (119–184)	0.03
HbA1c, %	6.5 (5.75–7.35)	7.4 (6.70–8.40)	0.007
Total cholesterol, mg/dL^c^	163.4 ± 35.1	194.5 ± 39.7	0.003
Albumin, g/dL^d^	3.77 ± 0.40	4.21 ± 0.41	0.0003
AST, U/L^e^	36.5 (25–46)	21 (18–28)	< 0.0001
ALT, U/L^f^	33.5 (22–53)	21 (15–31)	0.009
GGT, U/L^g^	89.5 (36–173)	27 (18–47)	0.001
Platelet count, 10^4^/µL^h^	18.3 (10.1–19.9)	21.3 (17.7–25.0)	< 0.0001
APRI^i^	0.534 (0.450–0.474)	0.275 (0.204–0.383)	< 0.0001
FIB-4^j^	2.33 (1.02–4.74)	1.47 (1.04–2.03)	< 0.0001

Data are expressed as mean ± standard deviation when approximately normally distributed or otherwise median (25th–75th percentiles) for continuous variables and number (percentage) for categorical variables

Data are missing for ^c^2, ^d^1, ^g^2, and ^h^12 patients with HCC, and ^a^412, ^b^132, ^c^268, ^d^324, ^e^31, ^f^30, ^g^101, ^h^153, ^i^158, and ^j^160 patients without HCC

*HCC* hepatocellular carcinoma; *AST* aspartate aminotransferase; *ALT* alanine aminotransferase; *BMI* body mass index; *GGT* gamma-glutamyl transpeptidase; *APRI* AST to platelet ratio index; *HbA1c* hemoglobin A1c

### Risk factors for HCC development

The HCC patients from the current study and those from the cohort study were combined after excluding overlapping cases (*N* = 4) (Appendix Fig. 7). Logistic regression analysis was performed to elucidate the risk factors for HCC development.

**Fig. 3:**
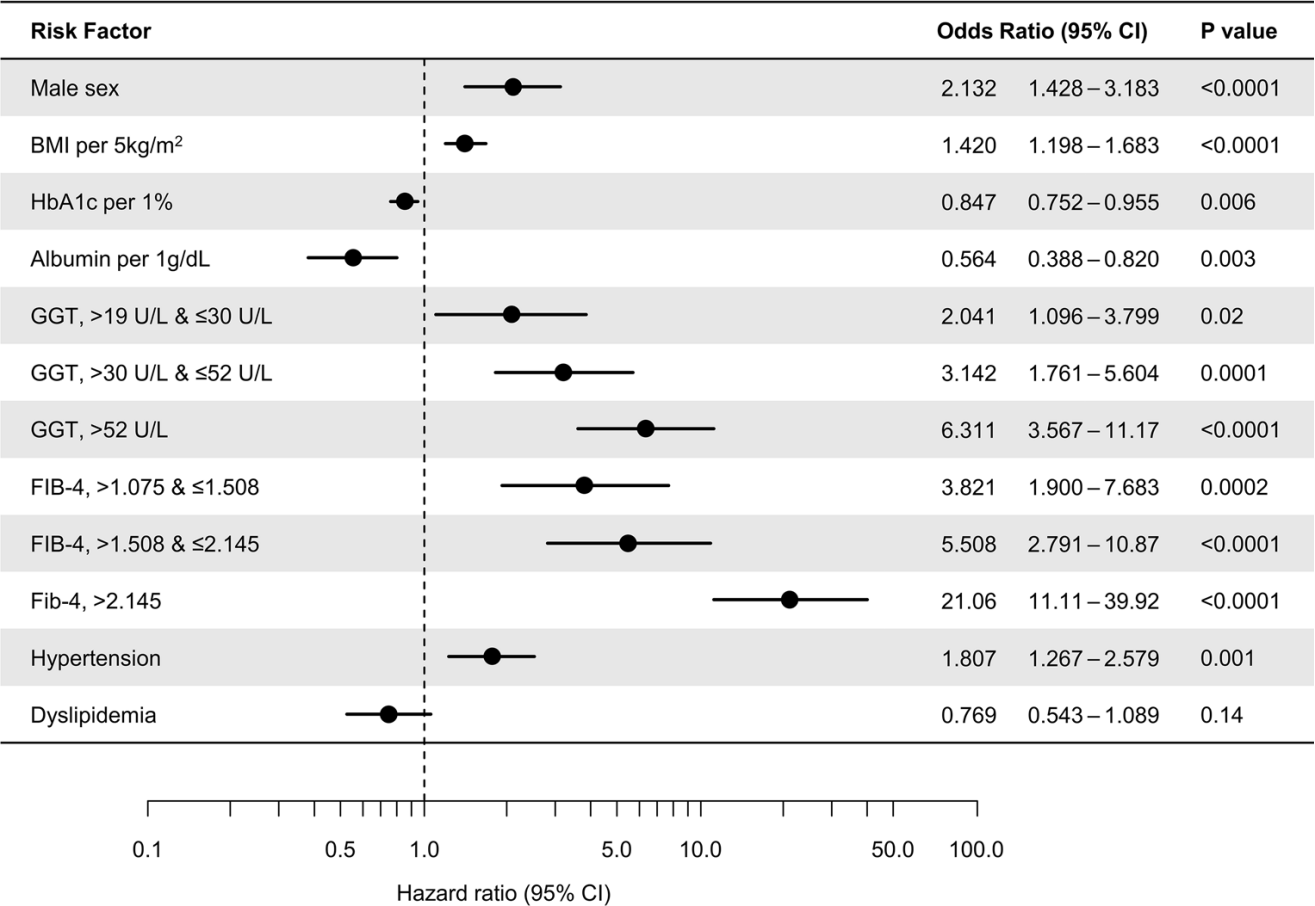
Multivariate logistic regression for HCC development (final model). The closed circles indicate point estimates of hazard ratios. The horizontal bars indicate 95% confidence interval. HCC, hepatocellular carcinoma

In univariate analyses, the following factors were associated with an increased risk of development of HCC: male sex, old age, higher BMI, AST, ALT, GGT, FIB-4 and APRI, lower platelet count, HbA1c, and albumin, and the presence of hypertension. To avoid multicollinearity, we evaluated several multivariate models. The results from the final model are shown in Fig. 3. Other tested models are shown in Appendix Table 4. Male sex, presence of hypertension, lower HbA1c and albumin, and higher GGT and FIB-4 were significant predictors of HCC development in diabetic patients. The presence of dyslipidemia was a negative risk factor with marginal significance.

### ROC analysis

**Fig. 4:**
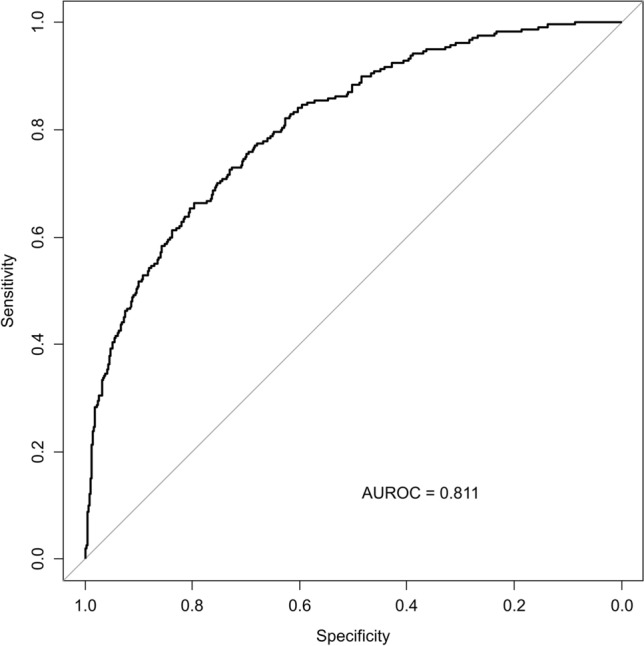
Receiver-operating characteristics (ROC) curve of FIB-4 for HCC development in diabetic patients. FIB-4, fibrosis-4; HCC, hepatocellular carcinoma

Since the FIB-4 index at entry showed a remarkable impact on HCC development, we performed an *ad-hoc* analysis to evaluate various cut-off values for FIB-4 and sensitivity, specificity, and positive likelihood ratio for the HCC incidence using ROC analysis (Fig. 4). When a cut-off value of 2.67 was applied, which was recommended as the best indicator for advanced fibrosis [31], the sensitivity was approximately 50%. We found that the cut-off value of 3.61 maximized the positive likelihood ratio, and the corresponding annual HCC incidence rate was estimated at 1.1% (Table 3). The result was robust after excluding those with a history of heavy alcohol drinking (≥ 60 g/day) (Appendix Fig. 8).

**Table 3:** Sensitivity, specificity, and positive likelihood ratio of annual HCC development according to cut-off values of FIB-4 index

FIB-4	Sensitivity	Specificity	Positive likelihood ratio	Estimated annual HCC incidence (%)
1.00	0.983	0.229	1.27	0.14
1.50	0.863	0.521	1.80	0.20
2.00	0.708	0.737	2.69	0.30
2.50	0.546	0.874	4.34	0.50
2.67	0.517	0.898	5.07	0.60
3.00	0.421	0.937	6.67	0.80
3.50	0.342	0.963	9.21	1.0
3.61	0.333	0.968	10.3	1.1
3.70	0.304	0.970	9.98	1.1
4.00	0.283	0.979	13.2	1.4
5.00	0.186	0.987	15.0	1.6

FIB-4, fibrosis-4; HCC, hepatocellular carcinoma

## Discussion

Diabetes is recognized as a well-established risk factor for HCC, however, other risk factors in diabetes patients without viral hepatitis have rarely been investigated so far because of the low incidence rate in the population [32]. In this study, we elucidated various potential risk factors for HCC, including male sex, old age, higher BMI, and higher FIB-4. The FIB-4 index, in particular, showed outstanding ability for extracting a high-risk group for surveillance.

Among various mechanisms that have been suggested as involved in the link between diabetes and hepatocarcinogenesis, insulin resistance and subsequent hyperinsulinemia, a key driver in metabolic syndrome, play a major role [33]. Nonalcoholic fatty liver disease (NAFLD), known as a hepatic manifestation of metabolic syndrome, and its progressive form, nonalcoholic steatohepatitis (NASH), are evident background conditions of HCC development [32]. However, the HCC incidence rate in NAFLD or NASH patients is as low as less than 0.1 per year unless the patients progress to cirrhosis [34–36]. Therefore, to identify patients with advanced fibrosis is mandatory for the surveillance of non-viral HCC. Besides, hepatic steatosis decreases according to disease progression, and in the final stage of the disease, known as burned-out NASH, fatty liver cannot be detected by regular ultrasonography [37]. The proportion of patients with fatty liver decreased during the follow-up period of this study. Therefore, the presence of fatty liver is not necessary for the enrollment of surveillance.

The FIB-4 index was originally developed for the prediction of significant fibrosis in patients with HIV/HCV coinfection [27]. Now, it is widely used for prediction of various liver diseases, including NAFLD [31]. Since the FIB-4 index includes age, there can be an argument that it overestimates liver fibrosis in elderly patients [38]. However, this disadvantage of the FIB-4 index in fibrosis estimation is rather an advantage in the risk estimation for HCC because old age is a strong risk factor in hepatocarcinogenesis. The APRI, another commonly used index for liver fibrosis, showed similar but slightly smaller predictive power than the FIB-4 index since the former does not include age as a component.

Although the specific protocol of surveillance for HCC in diabetes patients is open to debate, it would be a good idea to use the FIB-4 index as an entry test. Once-a-year examination of AST, ALT, and platelet count is inexpensive and acceptable. The cut-off value of 3.61 may identify patients at a 1% annual risk of HCC development. However, since the cut-off value misses approximately two-thirds of HCC patients, a two-step screening strategy combining FIB-4 and other non-invasive tests would be better. For that purpose, the cut-off value of 2.67 is a promising candidate. If the cut-off value of 2.67 was applied, approximately 10% of patients in the T2DM cohort would have undergone further examination.

In this study, higher BMI indicated an increased risk of HCC even after adjustment for other risk factors. Obesity, a key component of metabolic syndrome, causes adipose tissue remodeling and induces increased secretion of pro-inflammatory adipokines, including TNF-α and IL-6, and decreased anti-inflammatory adipokines like adiponectin, which potentially accelerates hepatocarcinogenesis [39, 40]. Obesity is reported to be a risk factor for HCC in both hepatitis B and C [41, 42].

Among key components of metabolic syndrome, whilst hypertension was an independent risk factor for HCC in this study, dyslipidemia was inversely related to HCC development, probably because of reduced cholesterol synthesis in advanced liver diseases. It should be noted that the absence of dyslipidemia along with decreased hepatic steatosis may not indicate a lower risk of HCC. Similarly, HbA1c superficially improved during the observation period of 5 years. This was mainly because the hemoglobin level also decreased due to the shortened half-life of erythrocytes according to cirrhosis progression. Thus, improved HbA1c values may also not indicate a decreased risk of HCC.

Excessive alcohol intake is an established risk for HCC [43]. In the present study, 16.3% of HCC patients and 27.8% of patients in the T2DM cohort who develop HCC were categorized as heavy drinkers (≥ 60 g/day), whereas the proportion was 4.4% in non-HCC patients. It is controversial whether heavy alcohol drinkers with T2DM should be treated within the same surveillance strategy for non-drinkers and moderate drinkers or outside the framework as a definite category of alcoholic liver disease. There is a concern that the FIB-4 index may not be useful for alcoholic liver disease in which AST elevation is more prominent than NAFLD. However, our previous reports of a nation-wide survey of non-viral HCC showed that the FIB-4 index was stable compared to AST and GGT during moderation in drinking [18].

The present study has several limitations. First, since the number of HCC cases in the cohort study was only 18, the annual incidence of HCC may not be statistically accurate. The cut-off points of the FIB-4 index, which was based on the incidence, should be validated in a prospective study. Second, this study could not elucidate the effect of anti-diabetic treatment on hepatocarcinogenesis. Metformin reportedly reduces the risk of HCC in diabetic patients [44]. We collected data regarding anti-diabetic treatment in this study. However, as metformin was regarded as contraindicated for cirrhosis and treatment choice for anti-diabetic drugs varies according to years, the statistical power was not enough to obtain a robust result. Third, as the HCC patients can be regarded as cancer-free for 5 years ahead of HCC diagnosis according to the enrollment criteria, the magnitude of risk factors for HCC development may be underestimated. Nevertheless, the risk factors that remained after multivariate analysis would be invariant. Forth, the prospective cohort consisted only of patients who were followed at the tertiary care hospitals. Since those who were referred to other affiliated hospitals and thus excluded from the prospective study might have a better condition, the cumulative HCC incidence could be overestimated.

In conclusion, we found various common risk factors for HCC development in diabetic patients. A simple calculation of the FIB-4 index in diabetes clinics can be the first step for the surveillance of HCC with non-viral etiology. We would start a prospective study to validate the efficacy of FIB-4-based surveillance strategy in the near future.

## Figures and Tables

**Fig. 5:**
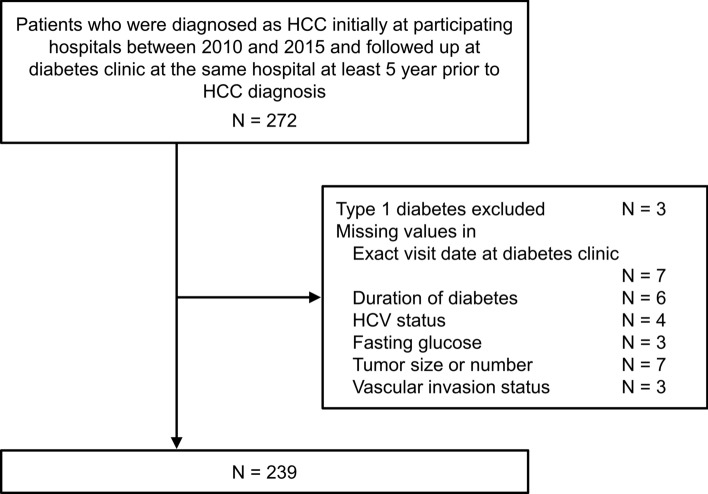
Patient enrollment flow: hepatocellular carcinoma patients at diabetes clinics

**Fig. 6:**
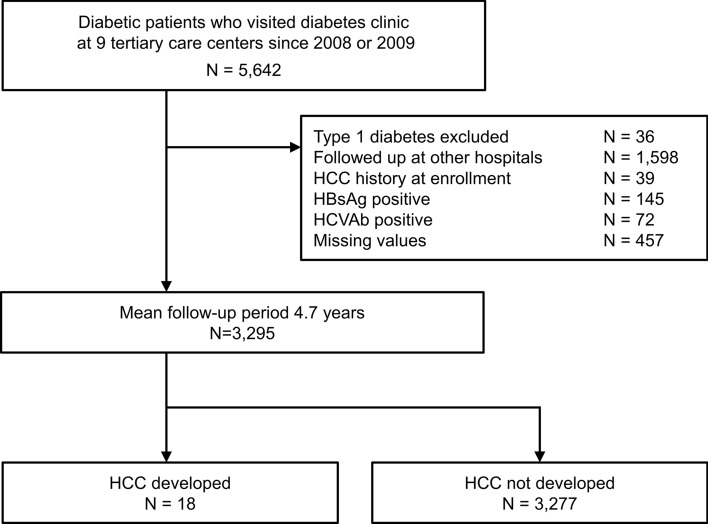
Patient enrollment flow: cohort study of diabetic patients

**Fig. 7:**
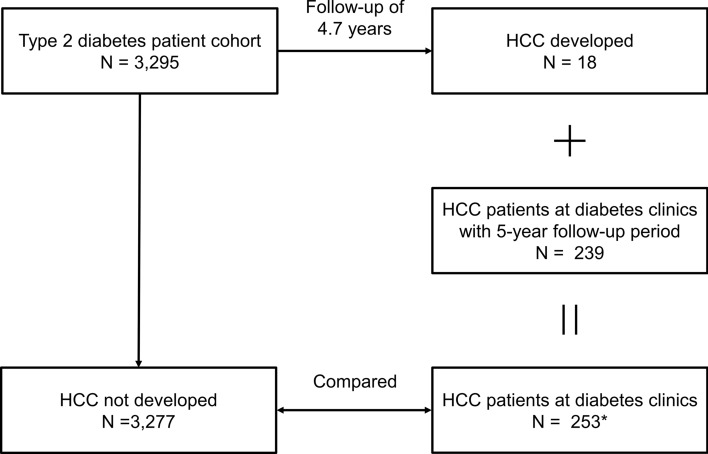
Patient enrollment: HCC cases and non-HCC cases were compared. * Overlapping cases (*N* = 4) were excluded

**Fig. 8:**
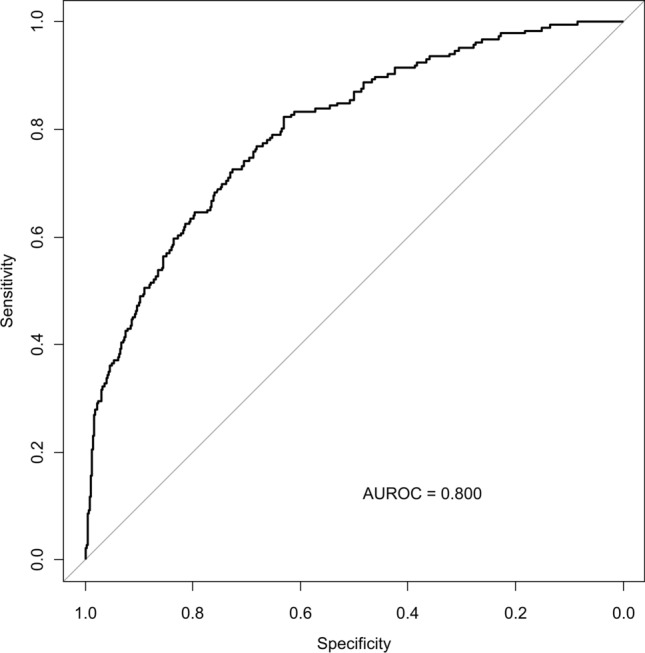
Receiver-operating characteristics (ROC) curve of fibrosis-4 for hepatocellular carcinoma: The subgroup analysis excludes those with a history of alcohol intake ≥ 60 g/day

## Appendix

See Figs. 5, 6, 7, 8 and Table 4.

**Table 4:** Univariate and multivariate logistic regression analyses

	Univariate analysis			Multivariate analysis: model 1			Multivariate analysis: model 2		
Variables	Odds ratio	95% confidence interval		Odds ratio	95% confidence interval		Odds ratio	95% confidence interval	
Male vs. female	2.50	(1.75, 3.56)	< 0.0001	2.07	(1.36, 3.14)	0.0006	2.40	(1.59, 3.62)	< 0.0001
Age, per 10 years	1.38	(1.20, 1.60)	< 0.0001	1.48	(1.22, 1.79)	< 0.0001	1.57	(1.31, 1.88)	< 0.0001
BMI, per 5 kg/m^2^	1.31	(1.14, 1.50)	0.0001	1.40	(1.16, 1.68)	0.0004	1.34	(1.13, 1.59)	0.0009
AST, IU/mL	1.03	(1.03, 1.04)	< 0.0001	1.02	(1.00, 1.03)	0.007			
ALT, IU/mL	1.01	(1.01, 1.02)	< 0.0001	1.00	(0.99, 1.01)	0.524			
Platelet count, 10^4^/µL	0.82	(0.79, 0.84)	< 0.0001	0.85	(0.82, 0.88)	< 0.0001			
HbA1c, %	0.86	(0.78, 0.96)	0.009	0.85	(0.75, 0.96)	0.008	0.83	(0.74, 0.94)	0.003
Albumin, g/dL	0.43	(0.32, 0.59)	< 0.0001	0.58	(0.39, 0.86)	0.007	0.45	(0.31, 0.66)	< 0.0001
GGT, U/L^a^			< 0.0001			< 0.0001			< 0.0001
≤ 19	1								
> 19 and ≤ 30	2.16	(1.20, 3.92)	0.011	1.93	(1.04, 3.60)	0.038	1.77	(0.95, 3.33)	0.074
> 30 & ≤ 52	4.00	(2.32, 6.90)	< 0.0001	2.91	(1.61, 5.24)	0.0004	2.20	(1.22, 3.98)	0.009
> 52	10.86	(6.43, 18.34)	< 0.0001	6.41	(3.50, 11.71)	< 0.0001	4.14	(2.28, 7.51)	< 0.0001
FIB-4^a^			< 0.0001						
≤ 1.075	1								
> 1.075 and ≤ 1.508	3.55	(1.79, 7.04)	0.0003						
> 1.508 & ≤ 2.145	5.42	(2.80, 10.49)	< 0.0001						
> 2.145	24.89	(13.54, 45.77)	< 0.0001						
APRI^a^			< 0.0001						< 0.0001
≤ 0.208	1								
> 0.208 and ≤ 0.287	2.28	(1.02, 5.10)	0.045				1.92	(0.84, 4.37)	0.120
> 0.287 and ≤ 0.412	7.79	(3.80, 15.98)	< 0.0001				5.27	(2.51, 11.05)	< 0.0001
> 0.412	29.63	(14.84, 59.16)	< 0.0001				17.3	(8.36, 35.80)	< 0.0001
Hypertension	2.08	(1.51, 2.86)	< 0.0001	2.08	(1.43, 3.02)	0.0001	1.78	(1.25, 2.55)	0.002
Dyslipidemia	0.74	(0.55, 1.00)	0.053	0.84	(0.59, 1.20)	0.336	0.76	(0.54, 1.08)	0.130

*BMI* body mass index; *GGT* gamma-glutamyl transpeptidase; *FIB-4* fibrosis-4; *APRI* aminotransferase to platelet ratio index

^a^GGT, APRI and FIB-4 were categorized according to the quartile points
